# Cold-tolerant phosphate-solubilizing *Pseudomonas* strains promote wheat growth and yield by improving soil phosphorous (P) nutrition status

**DOI:** 10.3389/fmicb.2023.1135693

**Published:** 2023-03-13

**Authors:** Hemant Dasila, V. K. Sah, Vandana Jaggi, Arun Kumar, Lakshmi Tewari, Gohar Taj, Sumit Chaturvedi, Kahkashan Perveen, Najat A. Bukhari, Tan Ching Siang, Manvika Sahgal

**Affiliations:** ^1^Department of Microbiology, Akal College of Basic Sciences, Eternal University, Rajgarh, Himachal Pradesh, India; ^2^Department of Microbiology, College of Basic Sciences and Humanities, G.B. Pant University of Agriculture and Technology, Pantnagar, India; ^3^Department of Agronomy, College of Agriculture, G.B. Pant University of Agriculture and Technology, Pantnagar, India; ^4^Department of Pediatrics and Human Development, Michigan State University, Grand Rapids, MI, United States; ^5^Department of Agronomy, Dr. Khem Singh Gill, Akal College of Agriculture, Eternal University, Rajgarh, Himachal Pradesh, India; ^6^Department of Molecular Biology and Biotechnology, College of Basic Sciences and Humanities, G.B. Pant University of Agriculture and Technology, Pantnagar, India; ^7^Department of Botany and Microbiology, College of Science, King Saud University, Riyadh, Saudi Arabia; ^8^School of Pharmacy, KPJ Healthcare University College, Nilai, Malaysia

**Keywords:** PSB, fertilizer, psychrotroph, principal component analysis, response surface methodology

## Abstract

It is well-known that phosphate-solubilizing bacteria (PSB) promote crop growth and yield. The information regarding characterization of PSB isolated from agroforestry systems and their impact on wheat crops under field conditions is rarely known. In the present study, we aim to develop psychrotroph-based P biofertilizers, and for that, four PSB strains (*Pseudomonas* sp. L3, *Pseudomonas* sp. P2, *Streptomyces* sp. T3, and *Streptococcus* sp. T4) previously isolated from three different agroforestry zones and already screened for wheat growth under pot trial conditions were evaluated on wheat crop under field conditions. Two field experiments were employed; set 1 includes PSB + recommended dose of fertilizers (RDF) and set 2 includes PSB – RDF. In both field experiments, the response of the PSB-treated wheat crop was significantly higher compared to the uninoculated control. In field set 1, an increase of 22% in grain yield (GY), 16% in biological yield (BY), and 10% in grain per spike (GPS) was observed in consortia (CNS, L3 + P2) treatment, followed by L3 and P2 treatments. Inoculation of PSB mitigates soil P deficiency as it positively influences soil alkaline phosphatase (AP) and soil acid phosphatase (AcP) activity which positively correlated with grain NPK %. The highest grain NPK % was reported in CNS-treated wheat with RDF (N–0.26%, P–0.18%, and K-1.66%) and without RDF (N-0.27, P-0.26, and K-1.46%), respectively. All parameters, including soil enzyme activities, plant agronomic data, and yield data were analyzed by principal component analysis (PCA), resulting in the selection of two PSB strains. The conditions for optimal P solubilization, in L3 (temperature-18.46, pH–5.2, and glucose concentration–0.8%) and P2 (temperature-17°C, pH–5.0, and glucose concentration–0.89%), were obtained through response surface methodology (RSM) modeling. The P solubilizing potential of selected strains at <20°C makes them a suitable candidate for the development of psychrotroph-based P biofertilizers. Low-temperature P solubilization of the PSB strains from agroforestry systems makes them potential biofertilizers for winter crops.

## 1. Introduction

Phosphorus (P) is not only an essential nutrient for plant growth but is also involved in diverse biochemical processes like lipid metabolism and the biosynthesis of cell membranes and nucleic acids (Ha and Tran, 2014). Despite its importance, P emerges as a limiting nutrient in global agricultural ecosystems (Lin et al., 2016). In plants, P plays an important role in regulating crucial physiological processes like photosynthesis and nutrient stress mitigation (Xiong et al., 2018). These properties of P as a nutrient make it an indispensable element that neither is substituted nor is replaced in agriculture. As per current research updates, the agriculture P requirement is approximately 0.1–0.5% of the total P concentration (Sharma et al., 2013). The soil P becomes unavailable to plants as it forms complexes with Al^3+^, Ca^2+^, and Fe^2+^ as phosphates (Vance et al., 2003; Siedliska et al., 2021). A large quantity of phosphatic fertilizers (P_2_O_5_) is added to restore soil for the available P deficit. This negatively affects the soil quality and puts an economic burden. Therefore, alternative methods for using P-based fertilizers economically without sacrificing soil quality are required to support sustainable agriculture. Since PSBs mobilize P to plants, which would otherwise remain deposited in the soil, the application of phosphate-solubilizing bacteria (PSB) becomes imperative. A century's worth of agricultural P would be supplied by the bacterial-mediated mobilization of free P from complex ore (Sharma et al., 2013). PSB comes under a broad category of potential plant growth-promoting rhizobacteria (PGPR) which provide nutritional value to plants *via* interaction with the rhizospheric part of the plant making them a possible candidate for biofertilizers. PGPR such as *Azospirillum, Bacillus, Enterobacter, Rhizobium, Serratia, Flavobacterium*, and *Pseudomonas* could act as possible biofertilizers. Bacteria can be utilized alone or in combination, for example, the application of consortium (combination of *Azotobacter* and PSB) can increase wheat crop output (Jain et al., 2021). Wheat is a winter crop and temperatures as low as 15°C are prevalent. The capacity of PSB to bind P in the soil microenvironment is affected by temperatures as low as 15°C. Screening PSBs that can promote P solubilization and plant growth and development even at low temperatures is therefore essential. As a result, utilizing plant-beneficial psychrotolerant bacteria offers an alternative strategy for increasing agriculture output by lessening the effects of low temperatures (Akhtar et al., 2016; Patni et al., 2018; Adhikari et al., 2021).

The most significant advantage of using these psychrotrophs is their ability to colonize cold habitats which are sometimes referred to as cold active microorganisms (CAMs). CAMs can be both psychrophilic and psychrotrophic. *Pseudomonas, Staphylococcus, Rahnella*, and *Stenotrophomonas* are a few significant agricultural bacterial groups that are CAMs (Vyas et al., 2010; Dolkar et al., 2018; Kadioglu et al., 2018; Araya et al., 2021). This has sparked research on new cold-tolerant bacterial species that are powerful PGPR and can assist reduce the cold effect on a crop in an agroforestry system to maximize crop yield (Kadioglu et al., 2018). To ameliorate soil P deficiency, inoculating the soil with psychrotroph PSB is a potential technique. *Pseudomonas* sp. has been used as a biofertilizer in early winter wheat varieties since it is a powerful psychrotroph (Selvakumar et al., 2013). PSB inoculation also minimizes reliance on chemical fertilizers (Alori et al., 2017). The production of organic acids (OAs) is one of the mechanisms involved in phosphorus (P) solubilization. OAs release P by solubilizing inaccessible organic and inorganic phosphorus and the most significant are gluconic acid, citric acid, lactic acid, and malic acid. *Pseudomonas* sp. is well-known to produce OA and is linked to its P solubilization ability (Kushwaha et al., 2020). As the agricultural output is positively correlated with soil health, therefore, recording variability in soil-health indicators after inoculation of biofertilizer is crucial for the assessment of the overall impact of this strategy. Soil microbial dynamics positively correlate with soil health (Jacoby et al., 2017), and one indicator of soil health is the activity of its enzymes (Dasila et al., 2022). The enzyme pool in the soil is directly influenced by the microbial population, which also directly impacts the availability of micronutrients- and macronutrients (Tahat et al., 2020). Since wheat is one of the most responsive crops toward fertilizers input, therefore, it is the model crop for PSB and plant interaction. The ability of low-temperature P solubilization of these PSB strains makes them a suitable candidate to test them as psychrotroph-based biofertilizers in field conditions. The potential of these PSB strains for plant growth-promoting activity has already been tested in pot conditions (Dasila et al., 2022). Low-temperature P solubilization adaptability of these PSB strains from agroforestry system makes them potential biofertilizers for winter crops. The present investigation also focused on the need of developing biofertilizers from agroforestry system as microorganisms from agroforestry system can be very useful tend in regulating the nutrient cycle due to rich soil organic matter.

## 2. Materials and methods

### 2.1. PSB collection

Four PSB strains, namely *Pseudomonas* sp.-L3 (Accession No. MG966341), *Pseudomonas* sp.-P2 (Accession No. MG966347), *Streptomyces* sp.-T3 (Accession No. MG966352), and *Streptococcus* sp.-T4 (Accession No. MG966353), were isolated from three *Dalbergia sissoo* Roxb. provenances in North region of Uttarakhand, India, located at Lachhiwala (30.2099°N latitude, 78.1342°E longitude**)**, Pantnagar (29.0369°N latitude and 79.4472°E longitude), and Tanakpur (29.0722°N latitude, 80.1066°E longitude), India (Joshi et al., 2019).

### 2.2. Phosphate and zinc solubilization index

*In vitro* phosphate and zinc solubilizing ability of four bacterial strains was determined using Pikovskaya's agar (Pikovskaya, 1948) and zinc (Zn) solubilizing agar medium (Sharma et al., 2012). In the Pikovskaya agar, the inorganic P source is tri-calcium phosphate (TCP) whereas in Zn solubilizing medium ZnO serves as an inorganic Zn source. A total of 20 μL of active culture from nutrient broth (NB) was spotted onto Pikovskaya and Zn agar plates and incubated for 5 days at 15°C at a low-temperature incubator. The halo zone appears around the bacterial colony which indicates a positive test for P and Zn solubilizing potential under *in vitro* conditions. The phosphate-solubilizing index (PSI) and zinc solubilizing index (ZSI) of four PSB strains were calculated **(**Saravanan et al., 2007).

### 2.3. Quantitative estimation of phosphorous and Fourier Transform Infrared Spectroscopy analysis

The quantitative inorganic phosphate-solubilizing potential of four PSB strains was assessed in the National Botanical Research Institute's Phosphate (NBRIP) medium containing glucose (10 g), tri-calcium phosphate-TCP (5 g), MgCl2 (5 g), MgSO4.7H2O (0.25 g), KCl (0.20 g), (NH4)SO4 (0.10 g), distilled water 1 (L), and the pH (7.0 ± 0.2). The log phase culture of four PSB strains was inoculated in NBRIP broth medium and incubated at 30 ± 2°C, 120 rpm in an incubator shaker for 5 days. Thereafter, cell-free supernatant was used to measure soluble P *via* spectroscopic analysis of reduced phosphomolybdic acid (blue color) by adding α-amino naptho sulfonic acid in the treated sample **(**Fiske and Subbarow, 1925) and comparing with P standard solution (100 mgL^−1^). At 5 DPI, prior to estimating P, the pH of bacterial cultures was monitored.

For organic acid detection, FTIR analysis was performed. The log phase culture of selected PSB strains was grown in NB medium and kept at 28 ± 2°C for 72 h in an incubator shaker. Then, the log phase culture of PSB strains was subjected to centrifugation (at 5,000 rpm, for 5 min), followed by filtration *via* a bacteriological filter (0.2 μM). An equal proportion of filtrate and ethyl acetate were mixed thoroughly for 20 min in a separating funnel, and after vigorous shaking, two layers were developed. The upper (ethyl acetate) layer containing the metabolites was separated, and the extract was concentrated by a rotator evaporator for analysis with FTIR (Sharma et al., 2016).

### 2.4. Consortium development

The compatibility of four PSB strains was tested *via* permutation and combination (Roshani et al., 2020). Freshly grown colony of each of the four PSB strains was inoculated in NB medium and incubated at 28 ± 2°C and 120 rpm for 24 h. The absorbance of bacterial suspension was taken at 600 nm. Finally, an equal culture volume of compatible PSB strains {A_600_-0.6} was dispensed to 100 mL of NB and thoroughly mixed to develop a PSB-based bacterial consortium (Prasad and Babu, 2017).

### 2.5. Seed bacterization and trial establishment

A superior wheat genotype UP 262 was obtained from the seed processing Center (SPC), G.B. Pant University of Agriculture and Technology, Pantnagar, India. The seeds were surface-sterilized with 1% sodium hypochlorite (NaOCl) solution followed by two/three times washing with sterile distilled water. The surface-sterilized seeds were then dried for 1 h in laminar followed by bacterization *via* immersing them in a log phase (10^7^ cells mL^−1^) liquid broth culture of four test PSBs and consortia (Tariq et al., 2007; Sharma et al., 2014). Thereafter, three seedlings hill^−1^ were manually transplanted in the field with a planting geometry of 15–20 cm followed by proper irrigation and monitoring under standard operating procedure (SOP). Field preparation for both experiments was started 1 week before the field setting. The field was plowed, and bunds of 15 cm in height were prepared. Each plot was 2 m long and 2 m wide having a net area of 4 m^2^. Approximately six lanes per plot were made and leveled carefully after seeds were set.

#### 2.5.1. Experiment field setup

**Figure 1:**
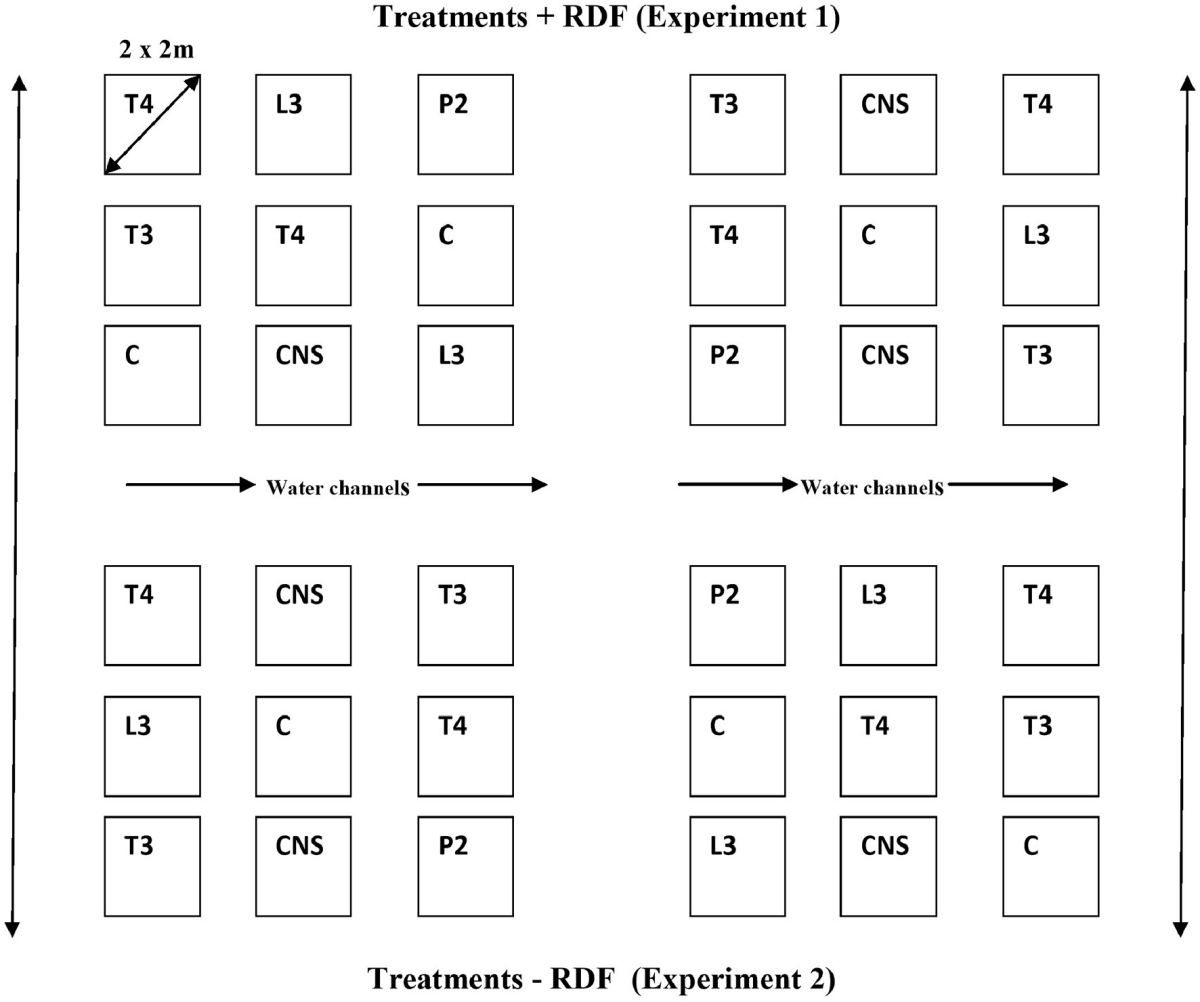
The detail of treatment in the field lay out showing two experiment sets, Experiment set 1 (Treatment + RDF) and Experiment set 2 (Treatment – RDF).

Two sets of the field were prepared. The set 1 (RDF + Treatments) and set 2 (RDF – Treatments) detailed information about the treatments is mentioned in Table 1. RDF used in field experiment 1 was N-80, P-40, and K-40 Kgha^−1^ with field design given in Supplementary Table 1. Field experiments were set up in randomized block design (Figure 1).

**Table 1:** Details of field trial with gross plot size.

**S.No**.	**Particular**	**Details**
1.	Design	Randomized block design
2.	Crop	Wheat (*Triticum aestivum*.)
3.	Variety	UP 262
4.	Number of replications per treatment	3
6.	Total number of plots	18 plots for field set I (treatment + RDF) and 18 plots for field set II (treatment-RDF).
7.	Gross plot size	2 × 2 m
8.	Net plot size	6 m^2^
9.	Spacing	20 × 20 cm

#### 2.5.2. Sampling of plants

Plant samples were carefully uprooted at 45 and 90 days post-inoculation (DPI) and analyzed for plant vigor parameters. Soil samples were also taken and analyzed for soil physico-chemical properties and soil enzyme parameters.

#### 2.5.3. Biometric assessment and yield growth parameters

Wheat samples (45 and 90 DPI) were subjected to biometric observation such as plant height, plant fresh weight, and number of tillers per plant. The grain yield (GY), biological yield (BY), 1,000 g wt, and harvest index (HI) were taken at the final harvesting. At final harvesting, the plants were dried for 72 h followed by thrashing. HI of each plot was calculated with the help of the following formulae:


$$\text{HI }=\;\frac{\text{Economic yield}}{\text{Biological yield}}\text{X 100}$$

### 2.6. Soil enzyme analysis

For soil health assessment, three soil enzyme activities, e.g., alkaline phosphatase (AP), acid phosphatase (AcP), and fluorescein diacetate (FDA) hydrolysis, were determined to evaluate the impact of PSB inoculation on soil health.

#### 2.6.1. Alkaline phosphatase activity

For AP activity, one g of soil, 250 μl of toluene, 4 mL of modified universal buffer (MUB), and 1 mL of 25 mM p-nitrophenyl phosphate (PNP) were added in a test tube and incubated at 37 ± 1°C for 2 h under gentle shaking condition. Calcium chloride (CaCl_2_) was added to stop the reaction and Tris buffer was added. The sample was then subjected to centrifugation followed by filtration through Whatman filter paper (0.2 μm) (Agri et al., 2022).

#### 2.6.2. Acid phosphatase activity

All the steps are the same as described in the determination of AP activity except pH which is adjusted to 5.4 for AcP and 7.4 for AP enzyme activity (Tabatabai and Bremner, 1969).

#### 2.6.3. Fluorescein diacetate hydrolysis

For FDA estimation, a tri-phenyl tetrazolium chloride (TTC) solution was used. Tris buffer of strength 0.1 M (pH −7.4) and TTC solution of 5 mL were added in a 100 mL flask containing 5 g of soil and incubated for 8 h. To stop the reaction, 25 mL of toluene was added followed by centrifugation at 4,000 rpm for 10 min. The supernatant collected from this was filtered, and the absorbance was read at 485 nm (Inbar et al., 1991).

### 2.7. Microbial diversity analysis on different media

The microbial population was measured in different media such as plate count agar (PCA) for total bacteria count, Ashby media for total nitrogen fixers, Aleksandrow media for potassium solubilizers, and Pikovskaya agar for P solubilizers, respectively. The soil samples were serial-diluted followed by pour plating, and then, plates were incubated for 2 days at 28 ± 30° C. Thereafter, bacterial colonies were counted (Messer and Johnson, 2000; Chai et al., 2015).

### 2.8. Soil physico-chemical properties

Soil physico-chemical properties (pH, EC, and available NPK) were analyzed before and after the final harvesting of the wheat crop. Soil pH and EC were determined according to the method of Bower and Wilcox (1965) and Jackson (1967), respectively. Organic carbon (OC) of soil was estimated by titration method using the standard protocol of Walkley and Black (1934). For soil, macronutrient estimation of nitrogen (N) was done according to the method of Hanway and Heidel (1952). For phosphorous (P) estimation, Hanway and Heidel (1952) protocol was used, and for potassium (K), Subbiah and Asija (1956) method was used.

### 2.9. N, P, and K status of grains

Wheat plants of both the field experiments, set 1 and set 2, were harvested, and their seeds were analyzed for N, P, and K content according to the method of Hanway and Heidel (1952) and Subbiah and Asija (1956), respectively.

### 2.10. Electrospray ionization mass spectroscopy analysis for organic acid detection

The organic phase of PSB strains (L3 and P2) was extracted as mentioned earlier in Section 2.3. The organic phase was further subjected to the direct ESI-MS using a Water Q-ToF micro mass spectrometer.

### 2.11. Wheat plant chlorophyll and carotenoid estimation

Chlorophyll a, chlorophyll b, and total chlorophyll (TC) of wheat plants were estimated *via* dimethyl sulfoxide (DMSO) method (Hiscox and Israelstam, 1979) where chlorophyll a, chlorophyll b, and TC were estimated by the following formulae:


$$\begin{array}{l} \text{ Chlorophyll a }({\text{mgg}}^{-\text{1}})\text{ = }\frac{\left(\text{12}.\text{7 }\times \;{\text{A}}_{\text{663}}\text{ + 8}.\text{02 }\times \;{\text{A}}_{\text{645}}\right)}{\text{1000 }\times \text{ W}}\times \text{ V}\\ \text{ Chlorophyll b }({\text{mgg}}^{-\text{1}})\text{ = }\frac{\left(\text{22}.\text{9 }\times \;{\text{A}}_{\text{645}}\text{+ 8}.\text{02 }\times \;{\text{A}}_{\text{663}}\right)}{\text{1000 }\times \text{ W}}\times \text{ V}\\ \text{Total chlorophyll }({\text{mgg}}^{-\text{1}})\text{ = }\frac{\left(\text{20}.\text{2 }\times \;{\text{A}}_{\text{645}}\text{+ 8}.\text{02 }\times \;{\text{A}}_{\text{663}}\right)}{\text{1000 }\times \text{ W}}\times \text{ V} \end{array}$$

The same procedure was followed for the estimation of carotenoid except that the absorbance of leaf extract was taken at 480 nm (Kirk and Allen, 1965). Carotenoid content was calculated by the following formulae:


$$\text{Carotenoid }({\text{mgg}}^{\;-\text{1}})\text{ = }\frac{\left[{\text{A}}_{\text{480}}\text{ + 0}.\text{11 }\times \;{\text{A}}_{\text{663}}\;-\text{ 0}.\text{638 }\times \;{\text{A}}_{\text{645}}\right]}{\text{1000 }\times \text{ W}}\times \text{V}$$

where W is the weight of leaf sample, and V is the volume of leaf extract.

### 2.12. Effect of carbon sources on P solubilizing activity

To analyze the impact of different C sources on the P solubilizing potential of two *Pseudomonas* strains L3 and P2 which were selected on the basis of plant vigor parameters upon vigorous statistical analysis. To study this, different C sources were used in the NBRIP medium. Log phase culture of L3 and P2 PSB strains were inoculated into NBRIP broth medium amended with 1% of glucose, fructose, maltose, and sucrose separately, uninoculated NBRIP medium serves as a control, and each treatment was done in three replicates. The quantity of solubilized P was measured using the ammonium molybdate method as discussed earlier.

### 2.13. Optimization of P solubilization potential on PSB strains using response surface methodology

P solubilization potential of PSB strain L3 and P2 was optimized using the RSM model in Design Expert software (Trial Version 10.0.6), *via* standardizing the effect of pH, temperature, and C source (glucose) concentration. To run this model, a central composite rotatable design (CCRD) including three variables with four central points was used. RSM model includes a base design that includes both upper and lower limits of the variable along with central points. 3D RSM plots were generated using a mathematical model (Firdous et al., 2017).

### 2.14. Principle component analysis

Principle component analysis was done to evaluate the overall response of *in vitro* and *in vivo* field results and further mapped them according to the response shared by them and finally resulted in the selection of potential PSB strains.

## 3. Results

### 3.1. PSI and ZSI potential of PSB strains

**Figure 2:**
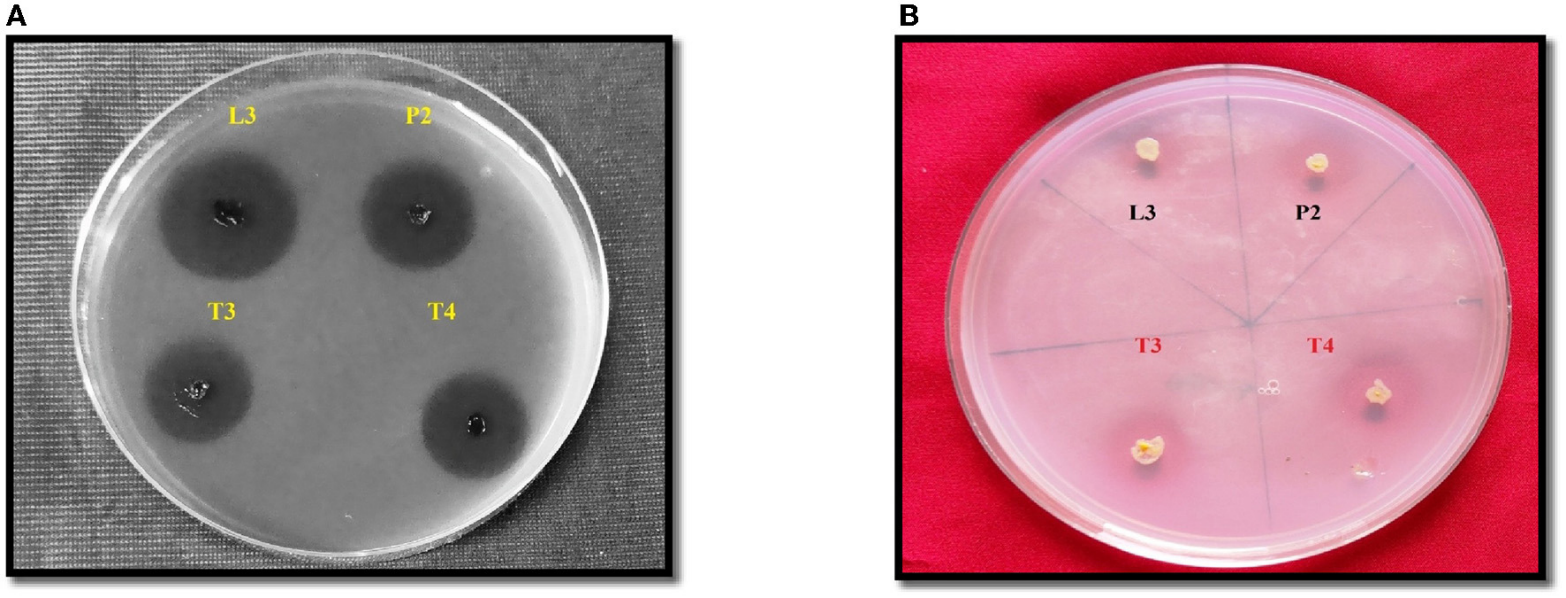
PSI **(A)** and ZSI **(B)** of L3, P2, T3 and T4 PSB, forming a clear halozone around the bacterial colony.

All four PSB strains tested positive for inorganic P solubilization. The maximum PSI response was observed in P2 (3.6 cm) followed by L3 (3.4 cm), T3 (2.7 cm), and T4 (2.4 cm). The four PSB strains also tested positive for Zn solubilizing potential, and maximum ZSI was observed in T3 (3 cm) followed by P2 (2.8 cm), T4 (2.6 cm), and L3 (1.8 cm) (Figure 2).

### 3.2. Quantitative estimation of P and FTIR analysis

**Figure 3:**
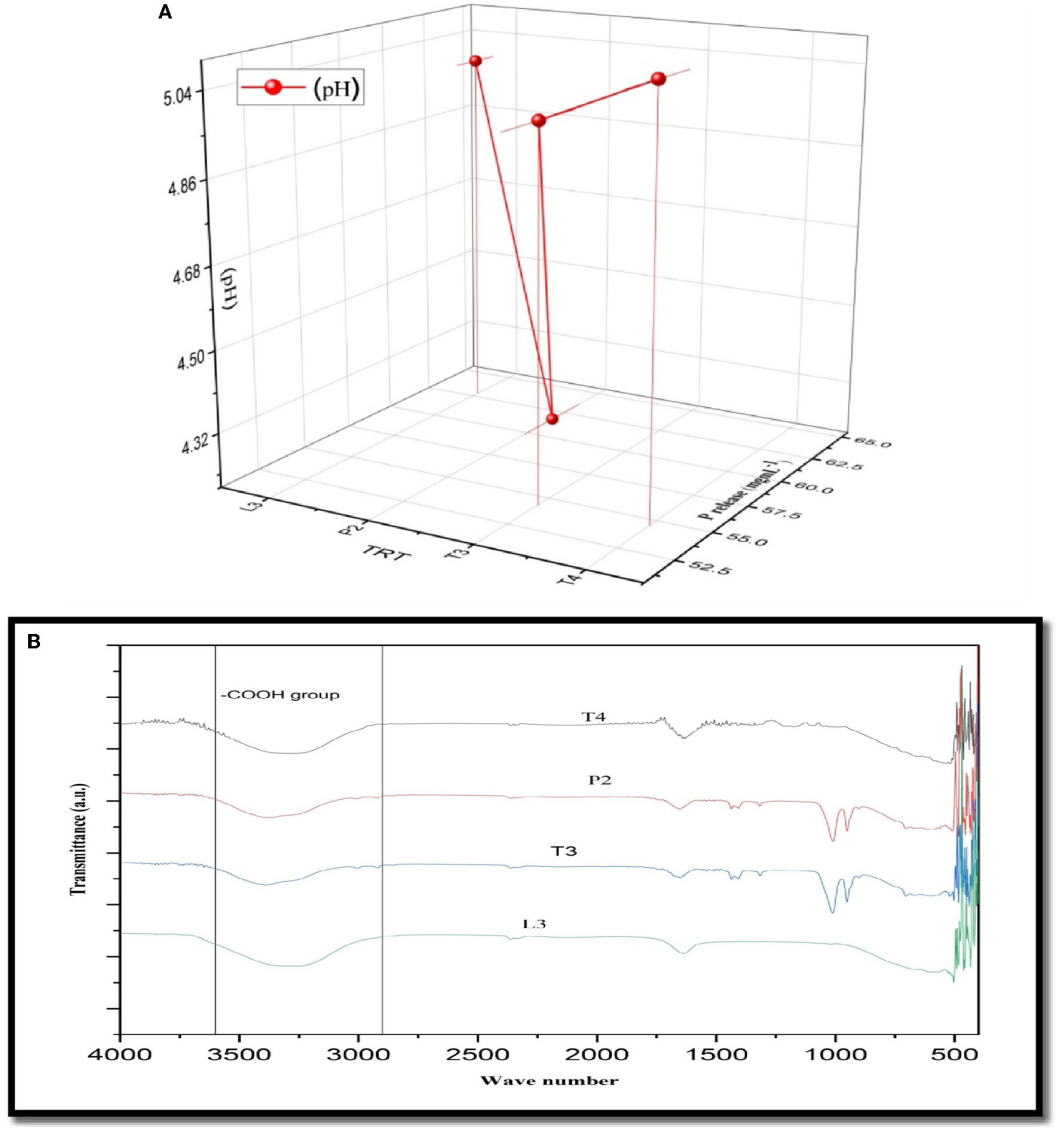
**(A)** Quantitative soluble P release along with pH drop (*r* = −0.52) in L3, P2, T3, and T4 PSB with correlation value. **(B)** FTIR analysis of PSB strains showing carboxylic group peak.

The amount of soluble P released in all four PSB strains was estimated in the NBRIP broth medium. Maximum soluble P was released in PSB strain L3 (62.83 ± 0.16^cd^ μgmL^−1^h^−1^) followed by P2 (Figure 3). In FTIR analysis, a peak of carbonyl bond was detected in all four PSB strains and it corresponded to the carboxylic group (Figure 3). Soluble P released for selected PSB strain (L3 and P2) at temperature 15 ± 2°C was 46.68 and 47.74 μgmL^−1^h^−1^, respectively. A significant amount of P has been solubilized even at this low temperature suggesting the cold adaptive solubilization potential of L3 and P2 PSB strain.

### 3.3. Consortium development

Out of four PSB strains tested, two strains L3 and P2 were compatible and used for the development of consortia.

### 3.4. Plant biometric and vigor parameters

Plant samples uprooted carefully during sampling (45 and 90 DPI) were used for recording agronomic traits.

#### 3.4.1. Field experiment set 1 (PSB + 100% RDF)

**Figure 4:**
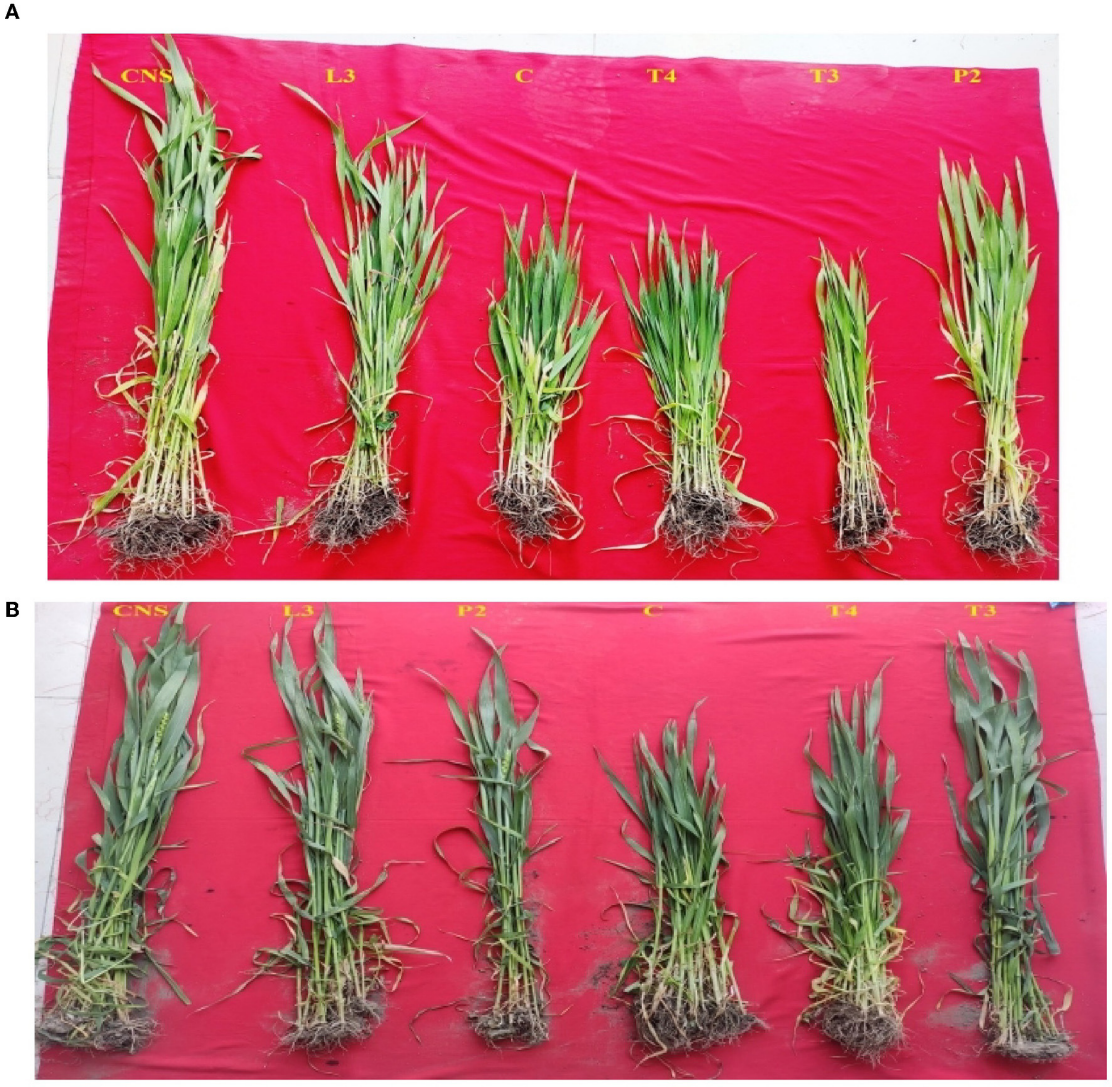
Variation among the treatment during sampling of field set 1 **(A)** and field set 2 **(B)**.

**Figure 5:**
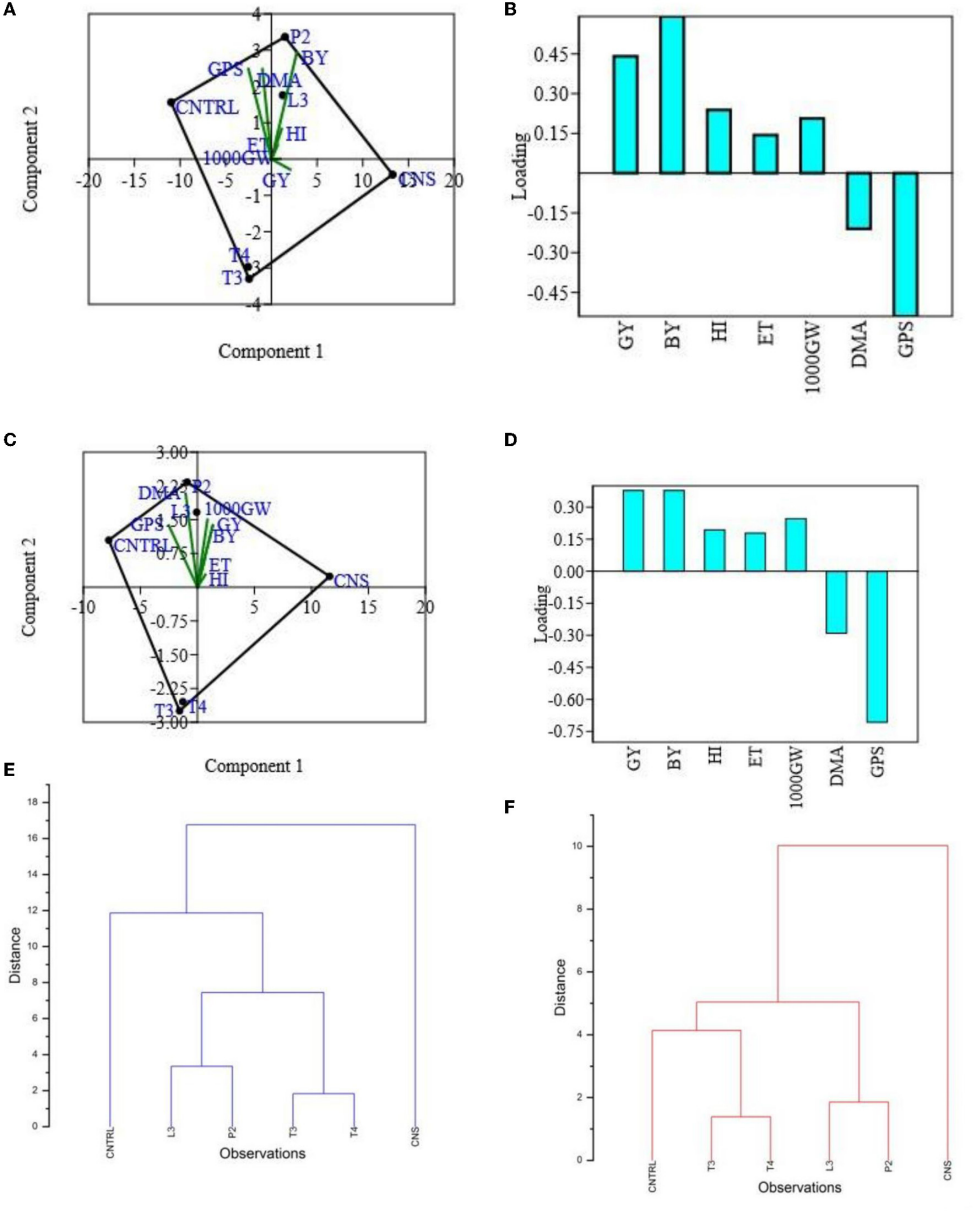
PCA **(A)** and loading plot **(B)** of plant vigor PSB treatments of type field set 1, PCA plot **(C)** and loading plot **(D)** of plant vigor PSB treatments in the field set 2, and hierarchal cluster analysis of PSB treatment in the field set 1 **(E)** and in the field set 2 **(F)**.

**Figure 6:**
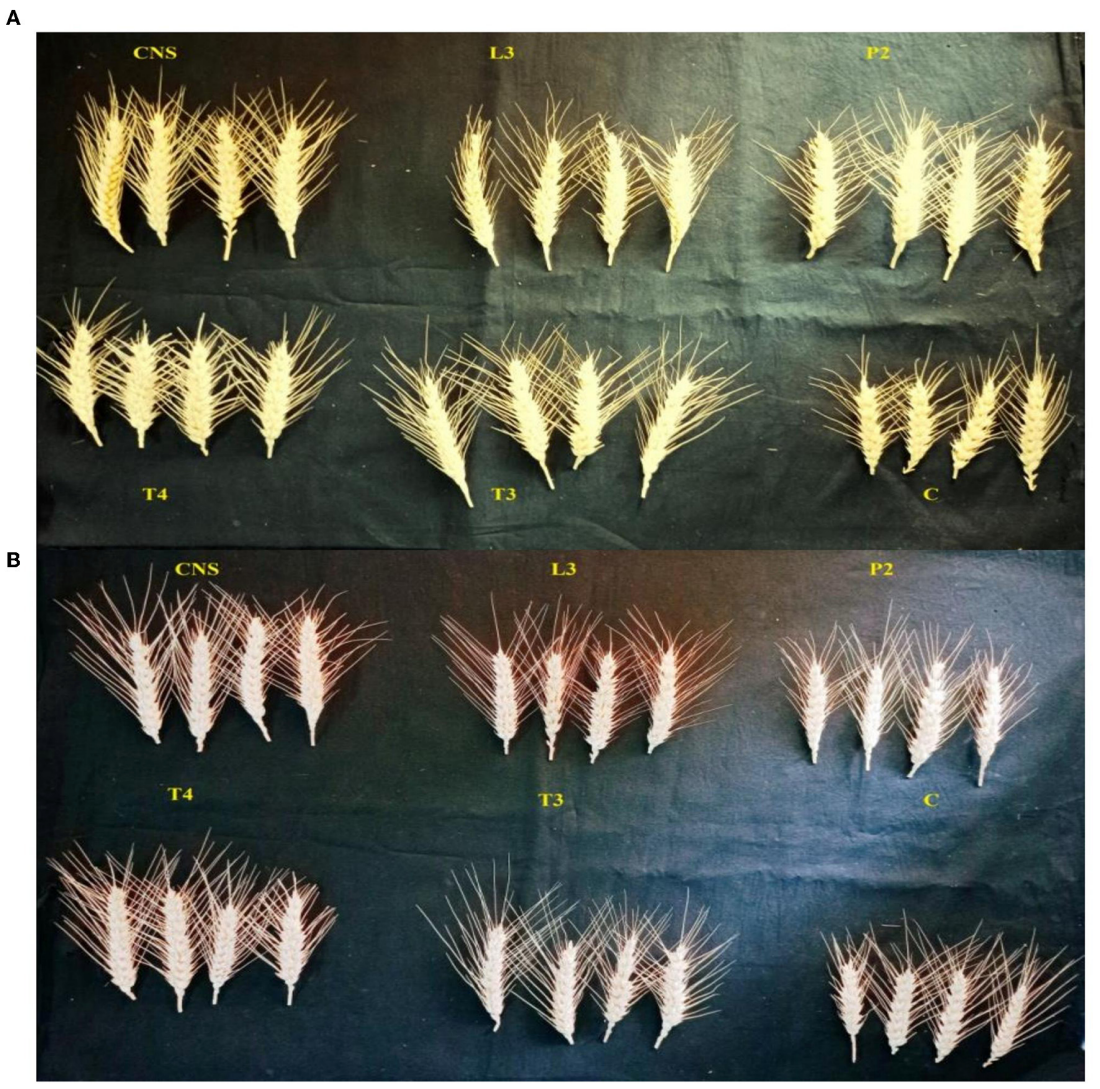
Grain per spike (GPS) variation in different PSB treatments in the field set 1 (PSB + RDF) **(A)** and field set 2 (PSB – RDF) **(B)**.

In the field experiment set 1, maximum agronomic response in terms of shoot length (SL), shoot fresh weight (SFW), root length (RL), and root fresh weight (RFW) was observed in CNS treatment followed by P2 treatment and agronomic response shared by them is significantly higher than the uninoculated control (C) (Table 2). Overall, there was a positive impact of inoculation of PSB in wheat plants as compared to uninoculated control (C) (Figure 4). The values of plant vigor parameters in the treated plant were significantly higher than in uninoculated control plants. The maximum response was observed in CNS treatment (Figure 6A) with GY (42.07 ± 0.6^e^), BY (105.84 ± 0.88^f^), HI (44.30 ± 0.08^d^), ET (12.16 ± 0.12^de^), 1,000 g wt (24.07 ± 0.21^d^), DMA (34.18 ± 0.18^d^), and GPS (36 ± 0.14^e^) being followed by P2 treatment (Table 3). In PCA loading plot, plant vigor parameters like BY and GY were found to be positively influenced upon PSB inoculation (Figures 5A, B). During cluster analysis, there were two out-groups, the first being CNS, the most effective, and the second out-group being C treatment, the least effective. Significant variation has been observed in GPS count among the PSB treatment (Figure 5E). There was a clear difference among the treatment in field conditions too (Supplementary Table 3).

**Table 2:** Agronomic parameters of PSB treatments with two different sets Set I (PSB + RDF) and Set II (PSB − RDF) at 90 days post-inoculation (DPI), respectively, where SL, shoot length; RL, root length; SFW, shoot fresh weight; RFW, root fresh weight.

	**Set I (PSB** + **RDF)**				**Set II (PSB** −**RDF)**			
**Treatments**	**SL**	**SFW**	**RL**	**RFW**	**SL**	**SFW**	**RL**	**RFW**
L3	78.64 ± 1.24^de^	8.25 ± 0.22^d^	7.3 ± 0.08^cd^	0.72 ± 0.01^de^	60.93 ± 0.41^cd^	4 ± 0.16^d^	5.1 ± 0.07^c^	0.44 ± 0.01^b^
P2	80.33 ± 1.63^e^	10.88 ± 0.35^e^	7.46 ± 0.11^d^	0.76 ± 0.01^de^	59.26 ± 0.65^cd^	3.53 ± 0.05^c^	5.25 ± 0.04^de^	0.45 ± 0.03^bc^
T3	72 ± 1.2^c^	6.5 ± 0.07^c^	5.87 ± 0.03^b^	0.5 ± 0.02^c^	53.4 ± 0.48^bc^	3.11 ± 0.08^bc^	4.73 ± 0.01^ab^	0.41 ± 0.01^a^
T4	64 ± 0.57^b^	5.93 ± 0.05^bc^	5.42 ± 0.02^b^	0.47 ± 0.08^ab^	54.73 ± 1.06^bc^	2.91 ± 0.07^bc^	4.75 ± 0.05^ab^	0.46 ± 0.08^bc^
CNS	83.33 ± 0.95^f^	9.38 ± 0.27^de^	8.4 ± 0.14^e^	0.82 ± 0.02^e^	62.66 ± 1.08^e^	5.42 ± 0.01^ef^	5.52 ± 0.03^e^	0.49 ± 0.06^c^
C	62.46 ± 0.86^ab^	5.4 ± 0.2^ab^	5.08 ± 0.2^a^	0.46 ± 0.06^ab^	45 ± 0.74^a^	2.01 ± 0.+	4.74 ± 0.1^ab^	0.41 ± 0.01^a^

Superscript alphabets in the values signify overlap of significance.

**Table 3:** Plant vigor parameters of wheat UP 262 genotype in all PSB treatments in FS 1 (field set 1) and FS 2 (field set 2), where FS 1 = PSB + RDF and FS 2 = PSB – RDF, GY, grain yield; BY, biological yield; HI, harvest index; ET, effective tiller; DMA, dry matter accumulation; GPS, grain per spike.

**Treatment**	**GY**		**BY**		**HI**		**ET**		**1,000 gwt**.		**DMA**		**GPS**	
	**FS 1**	**FS 2**	**FS 1**	**FS 2**	**FS 1**	**FS 2**	**FS 1**	**FS 2**	**FS 1**	**FS 2**	**FS 1**	**FS 2**	**FS 1**	**FS 2**
CNTRL	31.00 ± 0.46^a^	31.77 ± 0.21^a^	92.85 ± 0.13^ab^	64.64 ± 0.17^a^	38.84 ± 0.14^a^	30.64 ± 0.15^ab^	8.80 ± 0.16^ab^	6.06 ± 0.08^a^	19 ± 0.08^a^	15.03 ± 0.14^ab^	18 ± 0.14^a^	12.24 ± 0.07^ab^	22.00 ± 0.12^a^	10 ± 0.01^ab^
L3	38.84 ± 0.13^bc^	33.55 ± 0.14^bc^	98.90 ± 0.07^de^	82.26 ± 0.22^d^	41.06 ± 0.58^ab^	33.06 ± 0.18^cd^	11.31 ± 0.07^d^	8.72 ± 0.09^bc^	23.12 ± 0.4^bc^	18.60 ± 0.11^cd^	33.16 ± 0.58^cd^	13.86 ± 0.06^ab^	30.00 ± 0.18^bc^	11 ± 0.02^bc^
P2	39.05 ± 0.49^bc^	34.08 ± 0.26^bcd^	100.31 ± 0.95^e^	84.86 ± 0.28^cd^	42.59 ± 0.26^bc^	32.02 ± 0.34^bc^	11.01 ± 0.34	8.16 ± 0.07^de^	22.35 ± 0.21^bc^	19.30 ± 0.08^cd^	32.08 ± 0.22^bc^	14.36 ± 0.04^bc^	32.00 ± 0.49^cde^	12 ± 0.01^c^
T3	37.51 ± 0.09^c^	32.00 ± 0.19^b^	93.83 ± 0.14^ab^	70 ± 0.14^b^	39.8 ± 0.32^bc^	31.52 ± 0.26^ab^	9.77 ± 0.06^b^	7.14 ± 0.14^bcd^	22.08 ± 0.06^bc^	16.08 ± 0.12^b^	30.14 ± 0.18^bc^	14 ± 0.02^bc^	30.00 ± 0.09^cde^	10 ± 0.02^ab^
T4	36.72 ± 0.16^cd^	31.90 ± 0.28^a^	94.18 ± 0.35^cd^	72.12 ± 0.31^bc^	40.36 ± 0.45^c^	32.18 ± 0.16^bc^	10.21 ± 0.04^cd^	7.32 ± 0.16^bcd^	21.07 ± 0.45^ab^	16.74 ± 0.14^b^	30.00 ± 0.16^bc^	14.24 ± 0.04^bc^	30.00 ± 0.16^cde^	12 ± 0.02^c^
CNS	42.07 ± 0.60^e^	38.52 ± 0.27^d^	105.84 ± 0.88^f^	86.83 ± 0.32^e^	44.30 ± 0.08^de^	34.39 ± 0.25^cd^	12.16 ± 0.12^de^	9.60 ± 0.12^e^	24.07 ± 0.21^d^	19.96 ± 0.16^e^	34.18 ± 0.18^d^	16.64 ± 0.05^cd^	36.00 ± 0.18^e^	15 ± 0.03^cd^

Superscript alphabets in the values signify overlap of significance.

#### 3.4.2. Field experiment set 2 (PSB – 100% RDF)

The main purpose of PSB inoculation without RDF is to evaluate the potential of PSB strains on wheat plants with persistent inorganic fertilizer which was earlier applied in the soil. In the field experiment set 2 at 90 DPI, maximum agronomic response was observed in CNS treatment followed by L3 treatment. The response shared by them was significantly higher than the uninoculated control (C) (Table 2). Overall, the potential of PSB strains when applied alone was also found to be positive on wheat plants, and their response was quite significant from uninoculated control (C) (Figure 4). The influence on plant vigor parameters was maximum in CNS-treated plants with GY (38.52 ± 0.2^d^), BY (86.83 ± 0.14^e^), HI (34.39 ± 0.25^cd^), ET (9.6 ± 0.12^de^), 1,000 g wt (19.96 ± 0.16^e^), DMA (16.64 ± 0.05^cd^), and GPS (15 ± 0.03^cd^) being followed by P2 treatment (Table 3). The PCA of the plant vigor data also proved that the highest influence on plant variable was given by CNS followed by L3 and P2 in the order and GY was influenced the most (Figures 5C, D). Cluster analysis of plant vigor data of field experiment set 2 followed a similar pattern (Figure 5F). However, the overall response was low as compared to that in Field Experiment set 1, and GPS count was the most influenced parameter (Figure 6B).

### 3.5. Soil enzyme results

**Figure 7:**
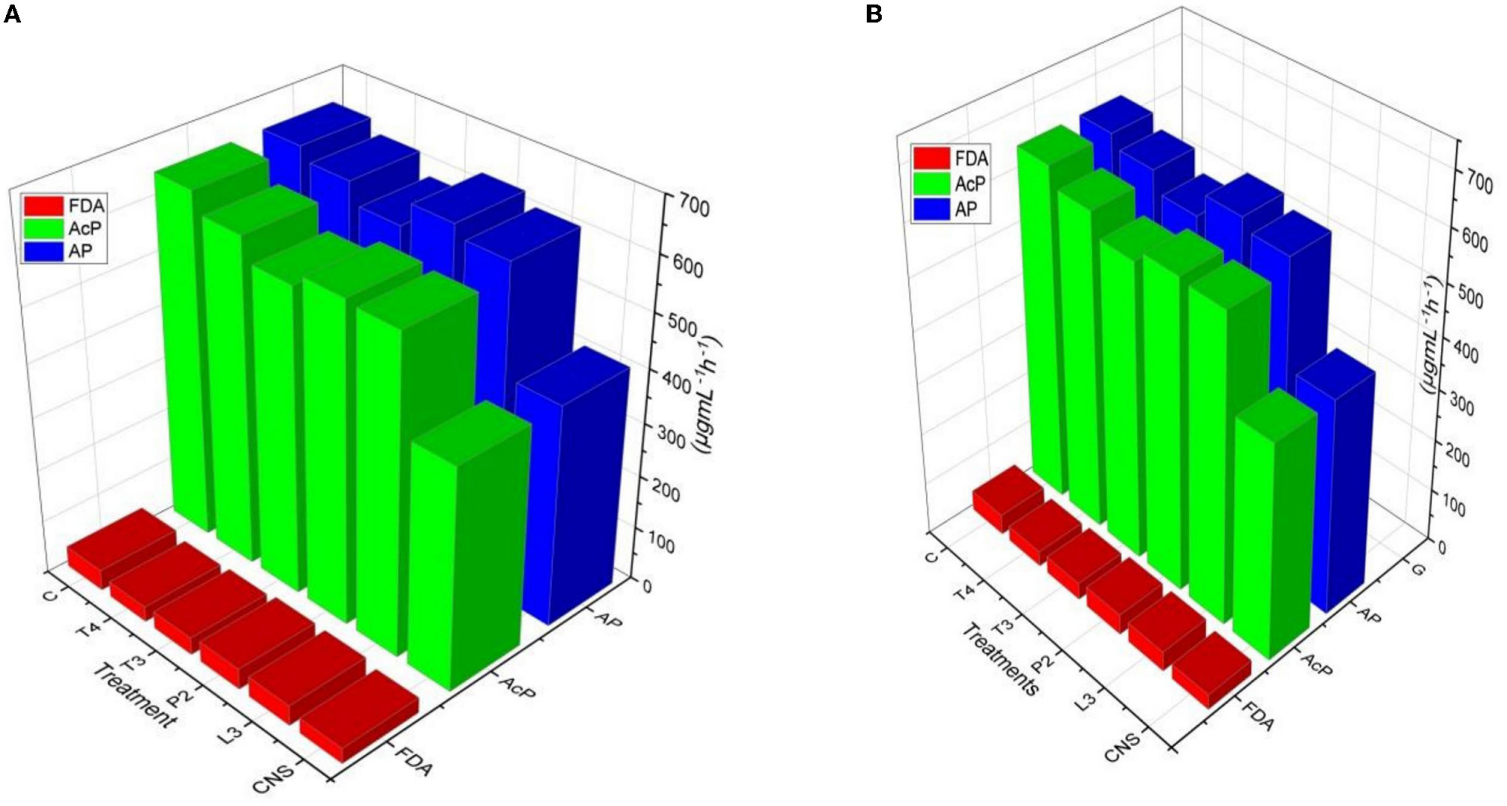
Soil enzyme variation includes FDA, AP, and AcP in response to PSB inoculation under field conditions in **(A)** Set I and **(B)** Set II after final harvesting.

Alkaline phosphatase and soil acid phosphatase soil enzyme activity represent the organic P conversion to soluble P. In the case of the field experiment set 1, maximum AP activity response was observed in CNS treatment (668.84 ± 0.23^f^) followed by T4 treatment (666 ± 0.18^ef^) which is quite significant from uninoculated control (C) soil (466 ± 0.12^a^ μgmL^−1^h^−1^). In soil AcP activity, the maximum response was observed in CNS treatment (662.62 ± 0.32^e^ μgmL^−1^h^−1^) activity. Soil AcP activity of uninoculated control-C (466 μgmL^−1^h^−1^) was found to be significant on the lower side. FDA represents the total soil microbial activity, and among treatments, the maximum response was observed in CNS treatment (48.06 ± 0.014^e^) followed by P2 (46.64 ± 0.08^de^ μgmL^−1^h^−1^) which were significant on the higher side as compared to uninoculated control-C (34.46 ± 0.16^a^) (Figure 7A). In a field experiment set 2, soil AP activity was maximum in CNS-treated soil (646.64 ± 0.24^f^ μgmL^−1^h^−1^) followed by P2 (632.24 ± 0.26^de^ μgmL^−1^h^−1^), and control (C) soil sample was found to be 412.68 ± 0.21^a^ μgmL^−1^h^−1^, respectively. In soil AcP activity, the maximum response was observed in CNS-treated soil (645.6 ± 0.18^f^ μgmL^−1^h^−1^) and T4 (610 ± 0.22^de^ μgmL^−1^h^−1^). In FDA activity in soil field experiment set 2, the maximum response was shown by CNS treatment (38.87 ± 0.13^cd^) and P2 (38.84 ± 0.18^cd^ μgmL^−1^h^−1^) (Figure 7B).

### 3.6. Microbial diversity analysis

**Figure 8:**
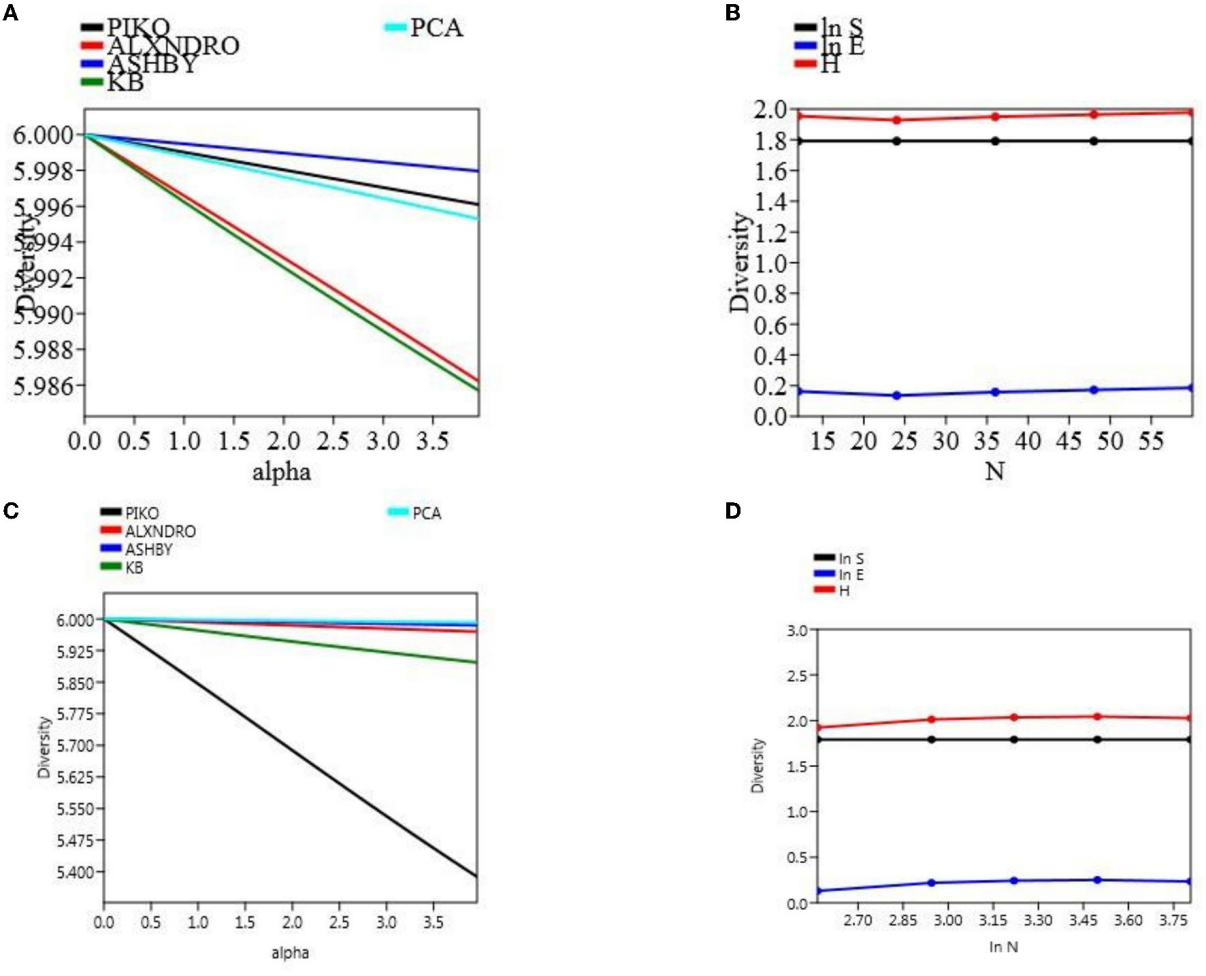
α-diversity indices of the microbial population with different media of field type 1 **(A)** and S.H.E indices of the field set 1 **(B)**. α-diversity **(C)** indices and S.H.E indices **(D)** of the field set 2.

The microbial count was taken out in different time intervals, and there was a clear significant difference among the population count, not only in two different field soil types but also within a treatment. Microbial CFU count on different media was taken at 10^4^ cells mL^−1^. At final harvesting in the field experiment set 1, maximum count was observed in soil treated with CNS (piko-2.55, alexandro-2.26, ashbyii-2.66, kings B-2.76, and PCA-2.86) which were significantly high as compared to uninoculated treated soil (piko-2.46, alexandro-2.2, ashbyii-2.56, kings B-2.48, and PCA-2.7). In a field experiment set 2, a similar pattern was observed in which maximum response was observed in soil treated with CNS (piko-2.12, alexandro-1.78, ashbyii-1.54, kings B-2.22, and PCA-2.66) and in uninoculated control (piko-2, alexandro-1.68, ashbyii-1.62, kings B-2.1, and PCA-2.54) (Supplementary Table 2). Alpha diversity of both field experiment set 1 (Figure 8A) and set 2 (Figure 8B) was measured, and S.H.E analysis was performed where S refers to species richness, H refers to hierarchal information, and E refers to evenness in a community (Figures 8C, D). S.H.E model was applied to check the goodness of fit among the population.

### 3.7. Soil physico-chemical analysis

The availability of soil OC, N, P, and K was enhanced in soils treated with PSB in both field experiments. In the field experiment set 1, the highest OC was found in CNS (0.88%)-treated plots followed by L3 (0.84%)-treated plots. However, in field experiment set 2, the highest OC was observed in L3 (0.78%)-treated plots followed by CNS (0.76%)-treated plots. Available N in field experiment set 1 was highest in CNS treatment (280.44 Kgha^−1^) followed by L3 treatment (274.64 Kgha^−1^), whereas in field experiment set 2, available N was maximum in P2 treatment (242.66 Kgha^−1^) followed by CNS (241.65 Kgha^−1^). Soil available P also varies with the treatments, and in the field experiment set 1, maximum P was found in CNS-treated soil (24.2 Kgha^−1^) followed by L3-treated soil (22.6 Kgha^−1^) and in field experiment set 2 too, a similar pattern was observed. Available K content follows the pattern of available P content. The pH and EC of various treatment soils are mentioned in Supplementary Table 3.

### 3.8. N, P, and K content of grains

**Figure 9:**
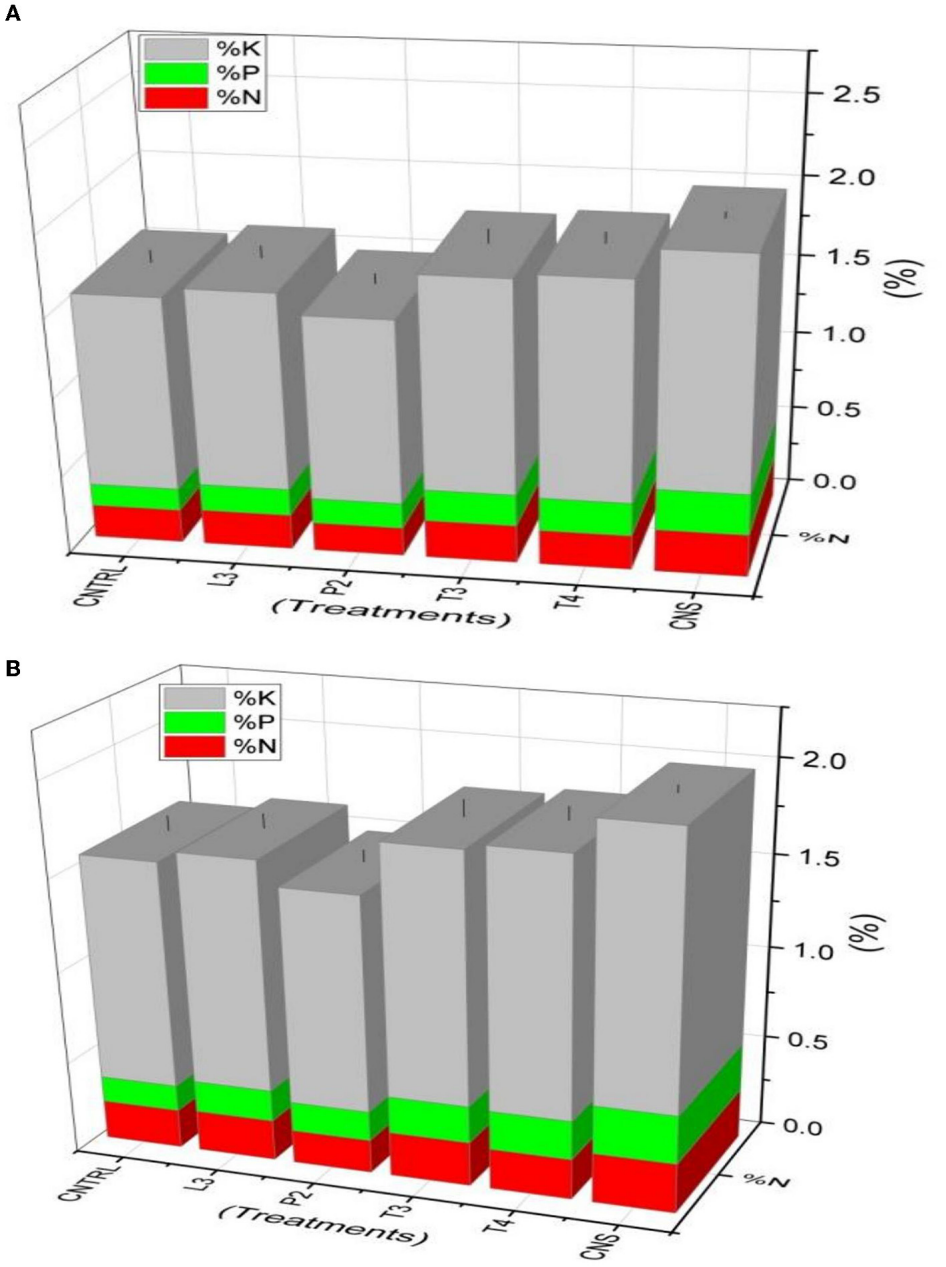
Nutrient analysis (N, P, and K) in plant sample **(A)** field set 1 (PSB + RDF), **(B)** field set 2 (PSB – RDF).

In the field experiment set 1 (PSB + RDF), maximum %N, %P, and %K content was found in CNS-treated wheat plants in which values were 0.35, 0.3, and 1.84, respectively, which were on the higher side of compared to uninoculated control (C) in which %N, %P, and %K were found to be 0.26, 0.18, and 1.66, respectively (Figure 9A). In field experiment set 2 (PSB – RDF), a similar pattern was observed where maximum %N, %P, and %K content was found in CNS with 0.27, 0.26, and 1.46%, respectively, which is significant from the control in which %N, %P, and %K were 0.21, 0.14, and 1.22, respectively (Figure 9B).

### 3.9. Chlorophyll estimation of wheat plants

**Figure 10:**
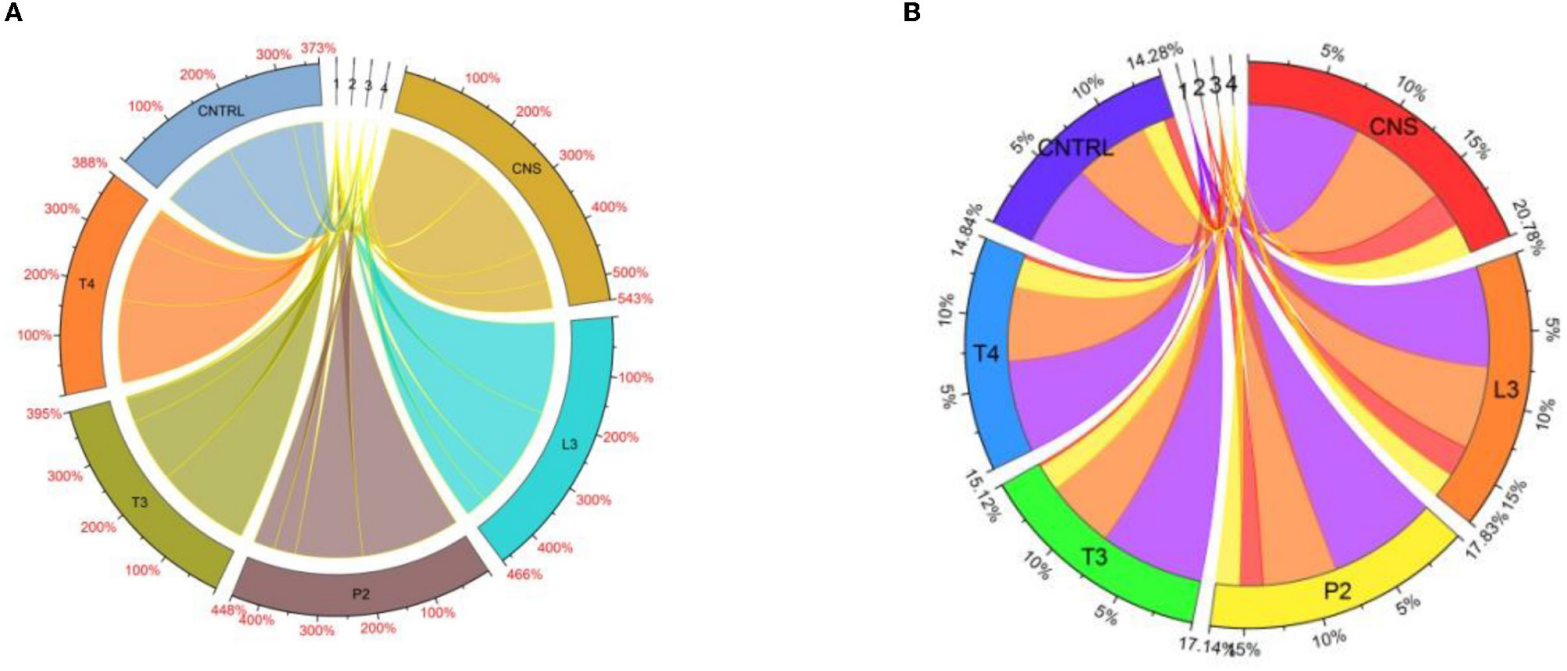
Chord analysis of chlorophyll showing response percent sharing of PSB treatments in the field set 1 **(A)** and field set 2 **(B)**.

Chlorophyll estimation of wheat plants directly links to nitrogen content present in a sample. In the field experiment set 1, maximum Chlorophyll A response was observed in CNS treatment (3.43), whereas in P2, T4, L3, and T3, it was found to be 2.76, 2.54, 2.33, and 2.33 mgg^−1^, respectively, which were on a higher side as compared to uninoculated control (2.12 mgg^−1^). For Chl B, the maximum response was observed in T4 (0.56 mgg^−1^)-treated wheat plants followed by P2 (0.35 mgg^−1^), T3 (0.28 mgg^−1^), L3 (0.18 mgg^−1^), and CNS (0.14 mgg^−1^) which are quite significant from uninoculated control (0.08 mgg^−1^). In the case of TC and carotenoid, the maximum response was observed in CNS-treated wheat plants which were found to be 2.31 and 0.6 mgg^−1^, respectively, which were significantly high as compared to uninoculated wheat plants in which TC and carotenoid content was found to be 1.72 and 0.49 mgg^−1^, respectively. In the field experiment set 2, chlorophyll A was maximum in CNS-treated (1.96 mgg^−1^) wheat plants, whereas, in L3, T4, P2, and T3, it was found to be 1.61, 1.45, 1.4, and 1.36 mgg^−1^, respectively. Most of the treatments, except T3, were significantly higher as compared to the uninoculated control (1.46). For Chlorophyll B, TC, and a carotenoid similar pattern was observed in which CNS-treated plants showed a maximum response and the CNS-treated values were 0.69, 2.18, and 0.59 mgg^−1^, respectively, which were significantly high when compared to their respective control in which the values of Chlorophyll B, TC, and carotenoid were found to be 0.19, 1.43, and 0.64 mgg^−1^, respectively. Chord analysis of chlorophyll and carotenoid data in both field experiment set 1 and experiment set 2 shows the maximum % increase share by CNS treatment (Figures 10A, B).

### 3.10. ESI-MS analysis of PSB strains

**Figure 11:**
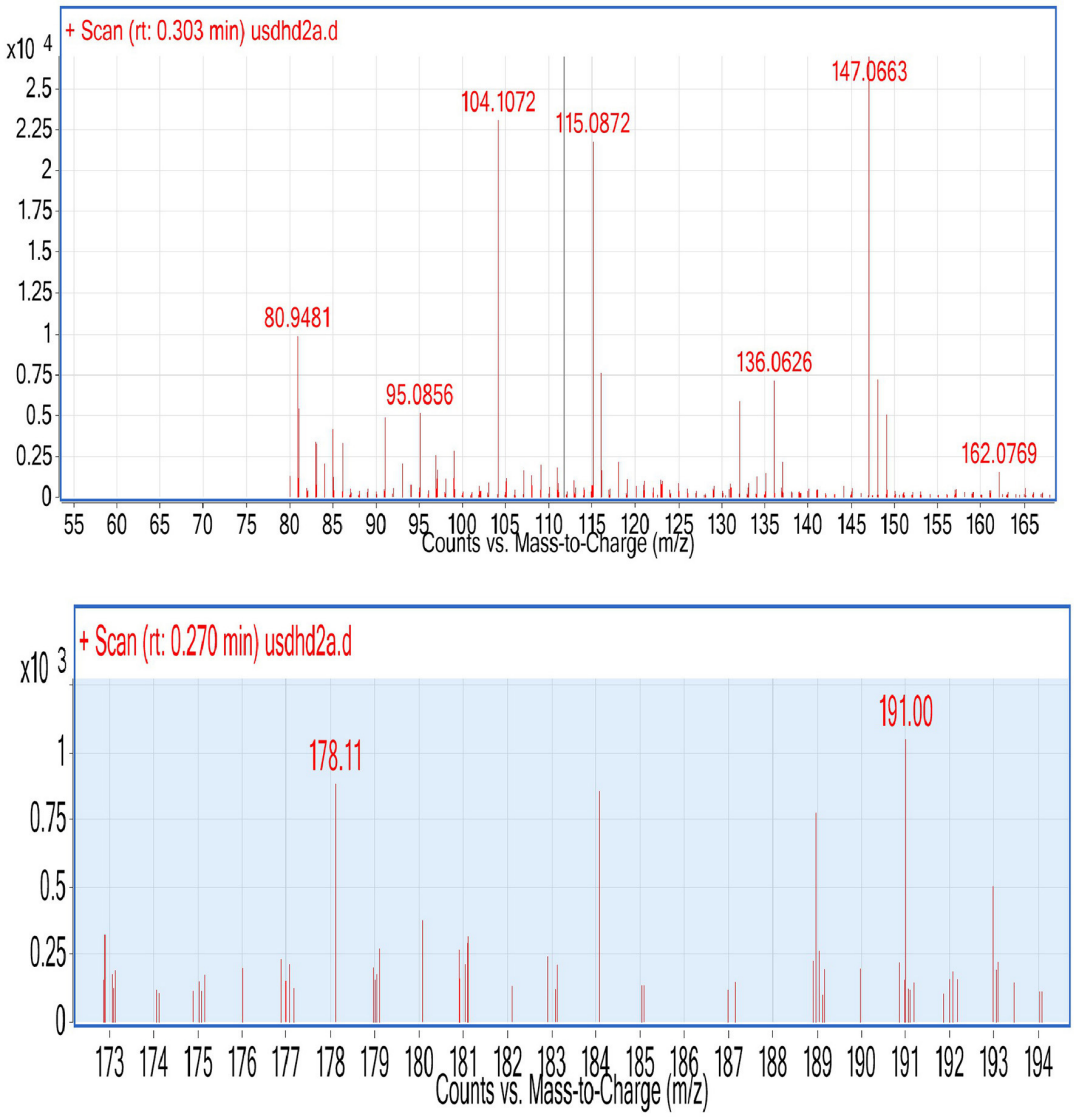
ESI (Electrospray ionization)-MS results of L3, P2, and CNS for the detection of organic acid.

During ESI-MS analysis, two organic acids, e.g., citric acid and maleic acid, were detected in selected L3 and P2 PSB strains, respectively (Figure 11). The production of OA has been considered one of the main mechanisms to solubilize inorganic P and these OAs have been reported earlier in studies.

### 3.11. Principal component analysis

Principal component analysis results revealed that CNS treatment is the most effective treatment in both the field condition (set I and set II) followed by L3 followed by P2 PSB treatments. These responses have been interpreted *via* plotting the performance of PSB treatments against plant vigor parameters in the component form which further results in selection of L3 and P2 PSB treatment (Figure 5).

### 3.12. Different C source effects on PSB solubilization

Maximum inorganic P solubilization in two PSB strains L3 (80 μg mL^−1^h^−1^) and P2 (78 μg mL^−1^h^−1^) was observed when glucose was used as a C source. P solubilization by two strains L3 and P2 in the following sugars is as follows: fructose (L3-74 and P2-72 g mL^−1^h^−1^), lactose (L3-62 and P2-64 g mL^−1^h^−1^), and sucrose (L3-68 and P2-64 g mL^−1^h^−1^), respectively.

### 3.13. Optimization of P solubilizing potential of PSB strains

**Figure 12:**
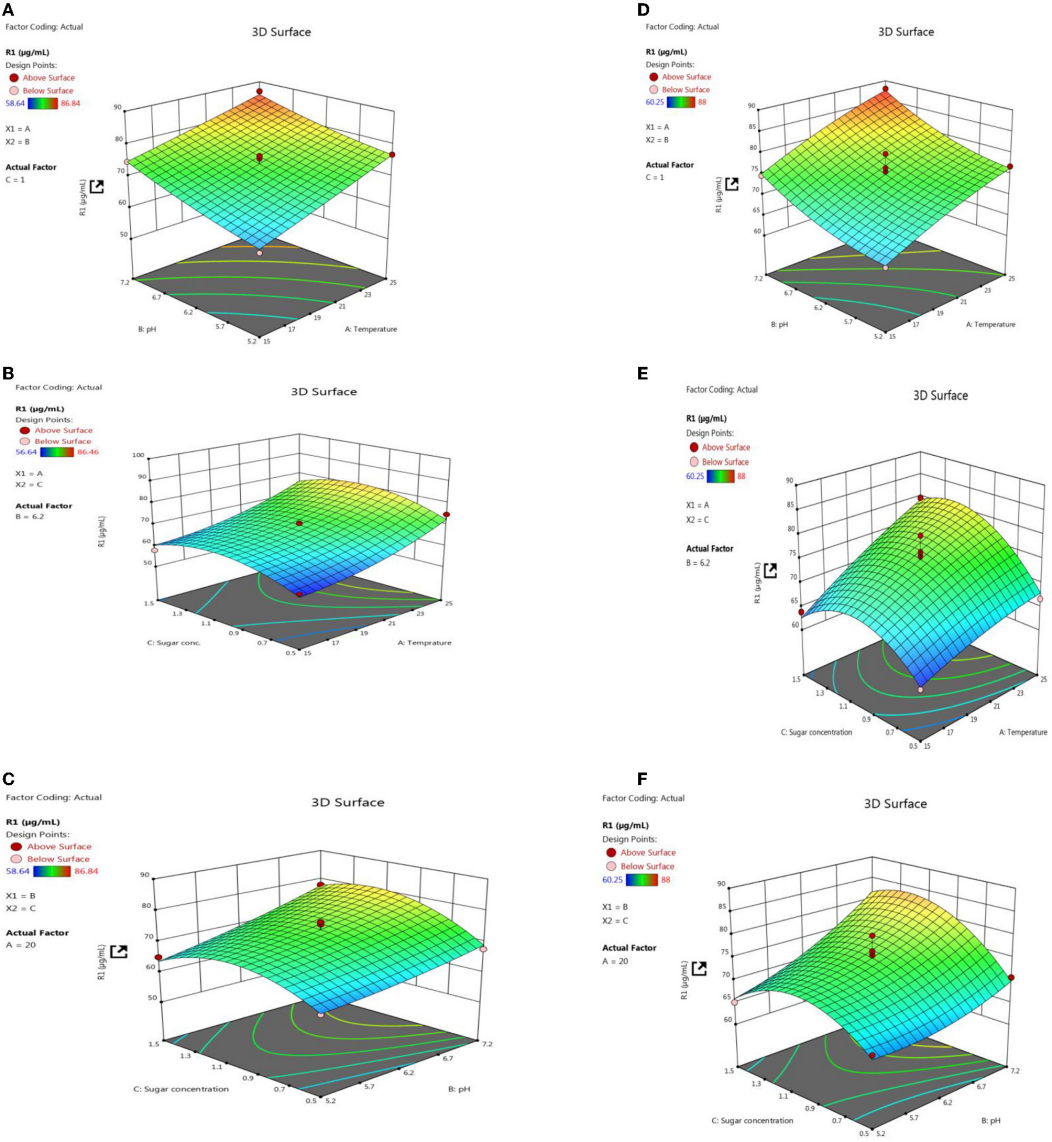
RSM plots for optimizing P quantification of L3 **(A–C)** and P2 **(D–F)** PSB treatment including the interaction of three variables, e.g., temperature, pH, and sugar concentration.

Response surface methodology plot of both PSB strains, e.g., L3 and P2, clearly indicates that quantitative P solubilization correlated positively with pH, temperature, and sugar concentration (Figure 12). However, the increase in P quantification was up to some points above that further increase in variable length decrease in P release. Most optimized condition for maximum P solubilization in L3 comes out to be at temperature-18.46°C, pH-5.2 with sugar concentration of 0.8% and for P2 optimized conditions are at temperature-17°C, pH-5.0 and with sugar concentration 0.89% respectively. However, a significant amount of P solubilization has been observed in both (L3 and P2) PSB strains even at low temperature and pH conditions which suggests that L3 and P2 PSB can be used as efficient P solubilizing psychrotroph.

## 4. Discussion

As phosphorous (P) is one of the major limiting factors in agriculture crop production, P solubilization *via* PSB provides a sustainable strategy to release the unavailable or precipitated soil P in an available form to plants (Messer and Johnson, 2000; Rawat et al., 2022). Although there is a high amount of total P content in the soil, its major portion is unavailable for plants. In this situation, the role of PSB alone or in combination (as a consortium) with inorganic or organic fertilizers as potent biofertilizers to release available P from soil to plants becomes prominent (Sharma et al., 2013). The present study established the role of potential PSB isolated from *the Dalbergia* forest ecosystem not only as CAM but also as PGPRs that enhance wheat growth under field conditions *via* influencing soil health and soil microbial communities positively when applied with RDF or without RDF. A major concern for any biofertilizer is its performance under field conditions. All four PSB were positive for *in vitro* P and Zn solubilization. However, maximum solubilization was in two *Pseudomonas* strains L3 and P2. The range of PSI and ZSI reported was similar to those reported in previous studies. Suleman et al. (2018) reported a PSI in the range of 3.2–5.8 cm for *Pseudomonas* sp., and Zhang et al. (2020) reported a PSI of 3.6 cm for *Pseudomonas lini* KM349410. The amount of P solubilized in the NBRIP broth medium ranges from 54.54 to 62.83 μgmL^−1^ with the strain L3 solubilizing maximum P (62.83 μgmL^−1^). Fahsi et al. (2021) reported that *Pseudomonas lini strain* KM349410 recovered from plant rhizosphere (*Jujube*) solubilized 69 μgmL^−1^ of inorganic P. The two strains L3 and P2 solubilized P at temperatures <20°C categorizing them as CAMs or psychrotrophic.

Organic acid production and protonation are two major mechanisms for inorganic P solubilization. P solubilization in L3 and P2 strains is associated with a pH drop. During P solubilization, the rate of protonation (H+) in the medium increases, and this could be the main reason for the pH drop. The pH in *Pseudomonas* strain P2 decreased to 4.2. A previous study reported a drop in pH (4.38) during P solubilization by a *Bacillus* strain (Upadhayay et al., 2022). There is an inverse relationship between soluble P and pH drop (*r* = −0.49) (Chen et al., 2012). Acidification *via* OA production is considered one most important mechanisms of P solubilization (Upadhayay et al., 2022). PSMs produce numerous organic acids that are reported to solubilize inorganic P in the medium including citric (pKa = 2.93 ± 0.28), oxalic (pKa = 1.38 ± 0.54), glycolic (pKa = 3.74 ± 0.11), and gluconic acids (pKa = 3.35 ± 0.35) (Halder et al., 1990; Akintokun et al., 2007; Patel et al., 2008; and Puente et al., 2009). In our study, two organic acids were detected during P solubilization *via* ESI-MS analysis (Figure 11), and these were maleic acid with an m/z value of 115.08 and citric acid with an m/z value of 191. These two organic acids might be responsible for P solubilization. Both OAs have been previously detected under P solubilizing conditions, e.g., malic acid (Patel et al., 2008) and citric acid (Puente et al., 2009). The production of carboxylic acids during p solubilization *via* acidification has been detected in FTIR analysis, for example, a peak corresponding to lactic acid was reported by Tang et al. (2019). Similarly, a broad peak of the carbonyl group mostly corresponding to the carboxylic group present in organic acid has been detected in four PSB strains (Figure 3B). A PSB inoculation promotes wheat development under field conditions. In both, the field experiment set 1 (PSB + RDF) and field experiment set 2 (PSB – RDF), agronomic response in PSB treatments was significantly higher as compared to the uninoculated control (C). Among the treatments, CNS treatment showed the maximum response in both field types. In field experiment set 1 (PSB + RDF), maximum SL was found in CNS treatment which is 0.33-fold higher than uninoculated control followed by L3 and P2 with 0.22- and 0.27%-fold increase, respectively. In the case of plant vigor parameters, the response of CNS treatment was maximum with a 0.13% increase in GY, 0.13 in BY, 0.14% in HI, 0.38% in ET, and 0.12% in 1,000 g wt. In addition to that, a 0.7% increase in SFW, a 0.66% increase in RL, and a 0.75 % increase in RFW were observed for control. In the field experiment set 2 (PSB – RDF), also maximum percent increase was observed in CNS treatment with 0.39% in SL, 1.69% in SFW, 0.16% in RL, and 0.21% in RFW. Plant vigor parameters in field experiment set 2 (PSB – RDF) were maximum in CNS treatment with 0.21% increase in GY, 0.06% in BY, 0.12% in HI, 0.58% in ET, and 0.32% in 1,000 g wt followed by L3 and P2 PSB treatments. The main factor that could be responsible for the maximum response of CNS is co-metabolism in which both PSB strains together (L3 and P2) metabolize or solubilize more complex compounds in the soil as compared to when used alone. This resulted in the greater mobilization of free available macro- and micronutrients and was consistent with the observation of Upadhayay et al. (2022), where treatment with consortia of strains having potential for both P and Zn solubilization showed maximum GY, BY, ET, and 1,000 g wt in rice plants. In our study performance of two *Pseudomonas* strains, e.g., L3 and P2 when used in combination (CNS treatment) and when used alone showed significant response as compared to uninoculated control, and these results were supported by the study where inoculation of *Pseudomonas* sp. MS16 resulted in increased wheat biomass and yield (Suleman et al., 2018).

The overall impact of PSB inoculation on soil health was evaluated by determining soil enzyme activities (AP, AcP, and FDA) post-inoculation. AP and AcP activities represent the conversion of organic bound P to the most available inorganic form to plants. In the field experiment set 1, maximum AP (66.64 μgmL^−1^h^−1^) and AcP (72.42 μgmL^−1^h^−1^) activity was found in CNS-treated soil which is significantly higher than in control where AP and AcP activity was 58 and 64.62 μgmL^−1^h^−1^, respectively. The observations were for field experiment set 2 (PSB – RDF). An increase in AP and AcP represents phosphatase activity and, thus, indicates the higher mobilization of free P in soil for utilization by plants. These results were also consistent with an earlier study where greater wheat biomass was accompanied by an increase in AP soil enzyme activity (Mäder et al., 2011). A similar pattern was observed for FDA activity too, FDA represents total microbial activity in the soil, and an increase in soil FDA activity could positively influence soil AP and AcP activity which in turn promotes plant growth. This explanation is supported by a previous experiment where a positive correlation between soil FDA and AP activity has been established for crop development (Dasila et al., 2018). Mobilization of P and other soil nutrients *via* PSB inoculation leads to better plant development.

To study soil microbial population dynamics, different media such as PCA (total bacterial count), King's B (for *Pseudomonas*), Pikovskaya agar (for positive phosphate-solubilizing bacteria), and Ashbyii (for N fixer) were used. The maximum α-diversity was found in PCA media and α-diversity among the treatment changes with soil status in both field experiment set 1 (PSB + RDF) and field experiment set 2 (PSB – RDF) as analyzed during S.H.E analysis (Figure 8). Microbial population dynamics change in our study has been supported by a previous study in which soil properties alter microbial population (Rfaki et al., 2018).

Soil physico-chemical properties such as OC, available N, P, and K content were maximum in CNS- and L3-treated soil in both field experiments. This observation provides an explanation that inoculation of PSB promotes soil nutrient status *via* solubilizing and mobilizing soil nutrients. The results of the present study were in tune with a previous study that reported an increase in soil OC content up to 1.79% upon PGPR inoculation (Shahdi Kumleh, 2020). In the present study, available soil and NPK content also increased as reported by Gupta et al. (2021), upon inoculation of *Pseudomonas aeruginosa* with a variable dose of fertilizers.

Plant nutrient status also changes with different treatments in both field types 1 and 2. In the field experiment set 1, % N, P, and K content in the field was maximum in CNS treatment with 0.35, 0.3, and 1.84%, respectively, which is significantly higher as compared to control in which % N, P, and K were found to be 0.26, 0.18, and 1.66, respectively. A similar pattern was observed in field experiment set 2. The main reason could be due to increased P and N availability in soil which is later utilized by the wheat plant itself for growth upon PSB inoculation. These results were supported by Elhaissoufi et al. (2020), where inoculation of PSB promotes wheat plant P content, and in another study, where PSB inoculation along with RDF in wheat plants promotes N, P, and K status in wheat plants (Sharma et al., 2013).

In both field experiment set 1 (PSB + RDF) and field experiment set 2 (PSB – RDF), there was a substantial increase in chlorophyll a, chlorophyll b, total chlorophyll (TC), and carotenoid content in the PSB-treated wheat plants, the and maximum was observed in CNS treatments (Figure 10). In the field experiment set 1 (PSB + RDF), chlorophyll a, chlorophyll b, total chlorophyll (TC), and carotenoid content in CNS treatment was 3.4, 0.14, 2.3, and 0.6 mgg^−1^, respectively, which is significant from control in which chlorophyll a, chlorophyll b, total chlorophyll (TC), and carotenoid content was 2.17, 0.08, 1.72, and 0.49, respectively. This may be due to the greater mobilization of soil P in wheat plants which later, in turn, promotes N content in plants (Schlichting et al., 2015; Adhikari et al., 2021), where mobilization of P causes an increase in chlorophyll and carotenoid content in wheat plants which positively correlated with N content in wheat plants. ESI-MS analysis of selected psychrotroph PSB strains L3 and P2 shows the production of two organic acids citric acid and maleic acid. P solubilization, *via* OA's production, is the principal mechanism adopted by PSB. Citric acid production by psychrotroph L3 PSB strain seems to have a prominent role in P solubilization and citric acid production by psychrotrophic *Pseudomonas* sp. which has also been previously reported, and in a similar manner, maleic acid is also reported to associate with P solubilization (Zaheer et al., 2019). Different C sources or sugars (glucose, fructose, lactose, and sucrose) were used to find out which C source supports maximum P solubilization in L3 and P2 PSB strain and that carbon source comes out to be glucose (L3-80 and P2-78). This could be because glucose is a primary carbon source and *Pseudomonas sp*. can use it *via* various metabolic pathways; these results are consistent with Suleman et al. (2018), in which *Pseudomonas* sp. MS16 solubilized more P in presence of glucose as a C source. Upon selecting glucose as a C source, the interaction effect of C source concentration, pH, and temperature on P solubilization potential was evaluated using RSM software. For both PSB strains, L3 and P2 conditions were optimized. Optimized conditions for both L3 and P2 PSB strains were found to be maximum at temperatures <20°C suggesting their application as biofertilizers in winter crops. RSM plots of both L3 and P2 PSB strains signify that the P solubilization rate increases with temperature and glucose concentration but decreases with an increase in pH. Upon analysis, it was found that temperature and glucose were positively correlated with P solubilization and pH is negatively correlated and these were supported by Suleman et al. (2018), a study in which similar observations were recorded.

Potential plant growth-promoting rhizobacteria including PSM play a significant role in P solubilization (Sharma et al., 2013), plant growth, and health promotion in various plants including wheat (Kalam et al., 2020; Kusale et al., 2021; Vafa et al., 2021; Ilyas et al., 2022). They have been seen as effective biostimulants (Hamid et al., 2021) and bioinoculants (Basu et al., 2021) under normal and cold stress conditions (Yadav et al., 2019; Baba et al., 2021). EPS-producing bacteria serve as potential biofloculant to mitigate cold stress (Sheikh et al., 2022).

Phosphate-solubilizing bacteria strains L3 and P2 have been submitted to the National Bureau of Agriculturally Important Microorganisms (NBAIM) culture collection with the following accession number *Pseudomonas paralactis* (L3)-NAIMCC-B-03170 and *Pseudomonas aeruginosa* (P2)-NAIMCC-B-03171, respectively.

## 5. Conclusion

To the best knowledge of our case study, this is the first report of PSB isolated from an agroforestry ecosystem (*Dalbergia sissoo* Roxb.) and their applications in winter wheat crops for growth and development under field conditions. The present study investigated the P solubilization potential of four PSB strains (L3, P2, T3, and T4) *in vivo* first followed by their application in wheat crops under field conditions. The response of all the PSB treatments in terms of plant agronomic and plant vigor parameters was higher as compared to the uninoculated control. PSB inoculation also promotes soil health and mitigates soil P into wheat plants *via* promoting NPK content in wheat seedlings, and later, two PSB strains (L3 and P2) and consortia were selected *via* exhaustive statistical analysis and used for further studies. Low-temperature growth and P solubilization of potential L3 and P2 make them suitable candidates for developing psychrotroph-based biofertilizers that can be used for winter crops. RSM plots also suggest the optimized P solubilization at a temperature <20°C for both L3 and P2 PSB strains.

## Data availability statement

The original contributions presented in the study are included in the article/Supplementary material, further inquiries can be directed to the corresponding authors.

## Author contributions

HD: writing and original draft preparation. MS: conceptualization, methodology, and supervision. VJ, VS, and LT: editing and reviewing of manuscript. GT: inputs for framing of manuscript. KP: reviewing of manuscript. NB and AK: validation of data. TS: reviewing of manuscript. All authors contributed to the article and approved the submitted version.
