# Supplementary material for: Cold-tolerant phosphate-solubilizing Pseudomonas strains promote wheat growth and yield by improving soil phosphorous (P) nutrition status
Source: Front Microbiol. 2023 Mar 13;14:1135693. doi: 10.3389/fmicb.2023.1135693 (PMC10072159; doi:10.3389/fmicb.2023.1135693)
Supplement: Supplementary file 1 [file Table_1.pdf]

Code	Treatments	Field set 1	Field set 2
**L3**	*Pseudomonas sp.*	L3 + RDF	L3 - RDF
**P2**	*Pseudomonas sp.*	P2 + RDF	P2- RDF
**T3**	*Streptomyces sp.*	T3 + RDF	T3- RDF
**T4**	*Micrococcus sp.*	T4 + RDF	T4- RDF
**CNS**	L3+P2	CNS + RDF	CNS- RDF
**C**	Uninoculated control	C + RDF	C- RDF

Table 1. Treatments details of PSB strains along with their NCBI accession number used in two parallel fields set 1 and set 2 respectively.
