# Supplementary material for: Cold-tolerant phosphate-solubilizing Pseudomonas strains promote wheat growth and yield by improving soil phosphorous (P) nutrition status
Source: Front Microbiol. 2023 Mar 13;14:1135693. doi: 10.3389/fmicb.2023.1135693 (PMC10072159; doi:10.3389/fmicb.2023.1135693)
Supplement: Supplementary file 2 [file Table_2.pdf]

| Treatment | PIKOVSKYAS* | | ALEXANDRO* | | ASHBY* | | K.B* | | PCA* | |
|---|---|---|---|---|---|---|---|---|---|---|
| | **F** | **U** | **F** | **U** | **F** | **U** | **F** | **U** | **F** | **U** |
| **L3** | 2.52 | 3.92 | 2.24 | 1.64 | 2.64 | 1.58 | 2.74 | 1.96 | 2.74 | 2.44 |
| **P2** | 2.48 | 2.88 | 2.34 | 1.74 | 2.6 | 1.54 | 2.72 | 1.88 | 2.76 | 2.52 |
| **T3** | 2.44 | 2.64 | 2.44 | 1.58 | 2.62 | 1.46 | 2.65 | 1.64 | 2.78 | 2.48 |
| **T4** | 2.42 | 2.55 | 2.3 | 1.54 | 2.58 | 1.48 | 2.64 | 1.86 | 2.7 | 2.56 |
| **CNS** | 2.55 | 2.12 | 2.26 | 1.78 | 2.66 | 1.54 | 2.76 | 2.22 | 2.86 | 2.66 |
| **CNTRL** | 2.46 | 2 | 2.2 | 1.68 | 2.56 | 1.62 | 2.48 | 2.1 | 2.7 | 2.54 |

**Table 2.** Population count after final harvesting in both field set 1(**F-**PSB+RDF) and field set 2 (**U-**PSB-RDF). (*population = x$10^4$ counts).
