# Supplementary material for: Cold-tolerant phosphate-solubilizing Pseudomonas strains promote wheat growth and yield by improving soil phosphorous (P) nutrition status
Source: Front Microbiol. 2023 Mar 13;14:1135693. doi: 10.3389/fmicb.2023.1135693 (PMC10072159; doi:10.3389/fmicb.2023.1135693)
Supplement: Supplementary file 3 [file Table_3.pdf]

Treatments	FS I (PSB+ RDF)				FS II (PSB - RDF)			
	OC (%)	N ($Kgha^{-1}$)	P ($Kgha^{-1}$)	K ($Kgha^{-1}$)	OC (%)	N ($Kgha^{-1}$)	P ($Kgha^{-1}$)	K ($Kgha^{-1}$)
**L3**	0.84	274.64	21.42	188.63	0.78	240.16	21	178.66
**P2**	0.71	268.63	22.6	178.56	0.72	242.66	20.8	176.56
**T3**	0.76	262.46	20.84	184.66	0.66	234.62	19.64	174.56
**T4**	0.78	262	21.64	180.26	0.68	238.66	20.84	172.28
**CNS**	0.88	280.44	24.2	192.06	0.76	241.65	21.68	174.54
**C**	0.66	236.54	20	176.68	0.6	232.46	18.68	170.2

Table 3. Effect of PSB on soil physico-chemical properties in two field set (FS) I and field set (FS II) 2 respectively, where OC= organic carbon, N=nitrogen, P=phosphorous and K= potassium.
