# Supplementary figures and images for: Cold-tolerant phosphate-solubilizing Pseudomonas strains promote wheat growth and yield by improving soil phosphorous (P) nutrition status
Source: Front Microbiol. 2023 Mar 13;14:1135693. doi: 10.3389/fmicb.2023.1135693 (PMC10072159; doi:10.3389/fmicb.2023.1135693)

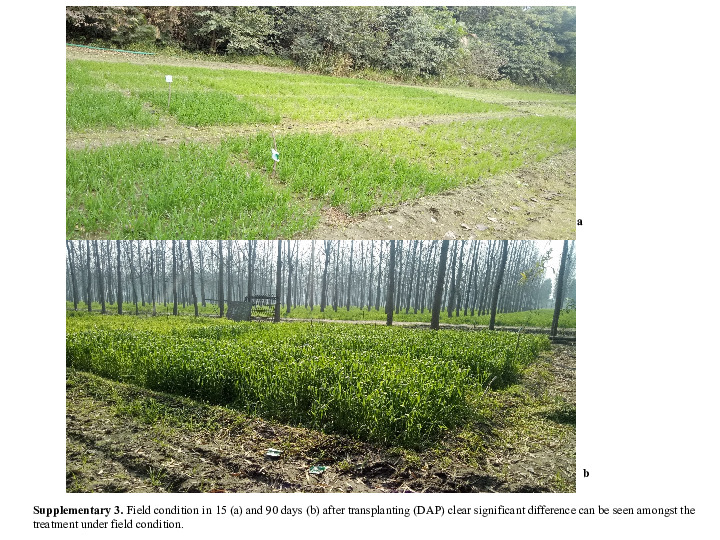

Supplement: Supplementary file 4 [file Image_1.jpg]
